# The Physicochemical Properties of Graphene Nanocomposites Influence the Anticancer Effect

**DOI:** 10.1155/2019/7254534

**Published:** 2019-07-03

**Authors:** Wenbo Yang, Xiangyu Deng, Wei Huang, Xiangcheng Qing, Zengwu Shao

**Affiliations:** Department of Orthopaedic Surgery, Union Hospital, Tongji Medical College, Huazhong University of Science and Technology, Wuhan 430022, China

## Abstract

Graphene nanocomposite is an inorganic nanocomposite material, which has been widely used in the treatment of tumor at present due to its ability of drug loading, modifiability, photothermal effect, and photodynamic effect. However, the application of graphene nanocomposite is now limited due to the fact that the functions mentioned above are not well realized. This is mainly because people do not have a systematic understanding of the physical and chemical properties of GO nanomolecules, so that we cannot make full use of GO nanomolecules to make the most suitable materials for the use of medicine. Here, we are the first to discuss the influence of the physicochemical properties of graphene nanocomposite on the various functions related to their antitumor effects. The relationship between some important physicochemical properties of graphene nanocomposite such as diameter, shape, and surface chemistry and their functions related to antitumor effects was obtained through analysis, which provides evidence for the application of related materials in the future.

## 1. Introduction

**Figure 1:**
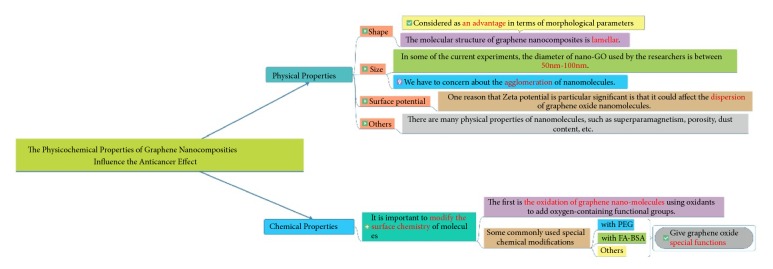
The idea of this article.

As a deadly disease, cancer has always caused a great threat to people's health [1, 2]. In the past few decades, the treatment of cancer has undergone great changes. At present, the treatment of cancer has formed a comprehensive treatment system combining surgery, chemotherapy, radiotherapy, and biological targeted therapy. However, all the above treatments have limitations. The five-year survival rate of patients with tumor is still very low. Clinical experience has shown that, with the exception of a few targeted drugs, most of existing therapies cannot improve the cure rate of cancer patients [3]. Taking chemotherapy as an example, most of the current chemotherapy drugs are nonspecific cell killers, which may cause the resistance of tumor cells to chemotherapy drugs and normal tissue cells damage [4–6]. Radiotherapy can also cause the damage of normal tissue cells [7]. Therefore, people are looking for more cancer treatment programs to improve the existing cancer treatment system. In recent years, nanomaterials are becoming more and more popular in antitumor researches. Graphene nanocomposites are representative among nanomaterials for their unique physicochemical properties [8]. So far, graphene-based materials have been synthesized in various forms, such as graphane (hydrogenated graphene), fluorographene (fluorinated graphene), graphdiyne, porous graphene, graphene nanoribbon (GNR), graphene oxide (GO), and reduced graphene oxide (rGO) [9]. Among them, GO and rGO are most commonly used for the biomedical application such as anticancer drug carrier, imaging agent delivery, and theranostics in oncology. GO is a highly oxidized form of graphene, which is often synthesized by the modified Hummer's method [10]. rGO is prepared by adding appropriate reducing agents to GO [11]. Therefore, both GO and rGO have the basic carbon planar structure of graphene and numerous *π*-*π* bonds, while water solubility is increased due to the presence of hydrophilic groups. At present, graphene materials are often used in the form of graphene nanocomposites for biomedical and clinical practice. Graphene nanocomposites with the basic structure of GO have some special effects of common nanomaterials, such as small size effect, which enables the materials not only to have an excellent photothermal effect to convert infrared light energy into heat energy effectively due to the special effects mentioned above and the presence of a large number of aromatic ring structure [12–16], but also to efficiently load aromatic hydrophobic drugs via hydrophobic interaction and *π*-*π* stacking [17, 18]. Graphene materials also have antibacterial properties [19], photodynamic effect [20], and so on. At present, it has been pointed out that the above characteristics of nanomaterials are closely related to the physical and chemical properties of nanomaterials [19, 21]. Our review aims to make a comprehensive summary of the relationship between the physical and chemical properties of graphene nanocomposites and their related characteristics in oncotherapy, so as to provide evidence for the application of graphene nanocomposites in the future. The overall idea of the article is shown in Figure 1.

## 2. Effects of Physicochemical Properties of Graphene Nanocomposites on Their Antitumor Activity

### 2.1. Physical Properties of a Material Molecule

Graphene nanocomposites have a variety of special properties, such as photothermal effect, photodynamic effect, and drug loading effect. Whether these properties can be successfully applied to the practice of eliminating tumor cells is closely related to the physical properties of the material molecules. At present, we pay more attention to the physical properties of material molecules. In the following part, some basic and important physical properties of graphene oxide nanocomposites currently in use and research such as shape, size, and surface potential will be introduced in detail, and the influence of these physical properties on the antitumor use of nanomaterials will be discussed.

#### 2.1.1. The Shape of GO Nanoparticles

**Figure 2:**
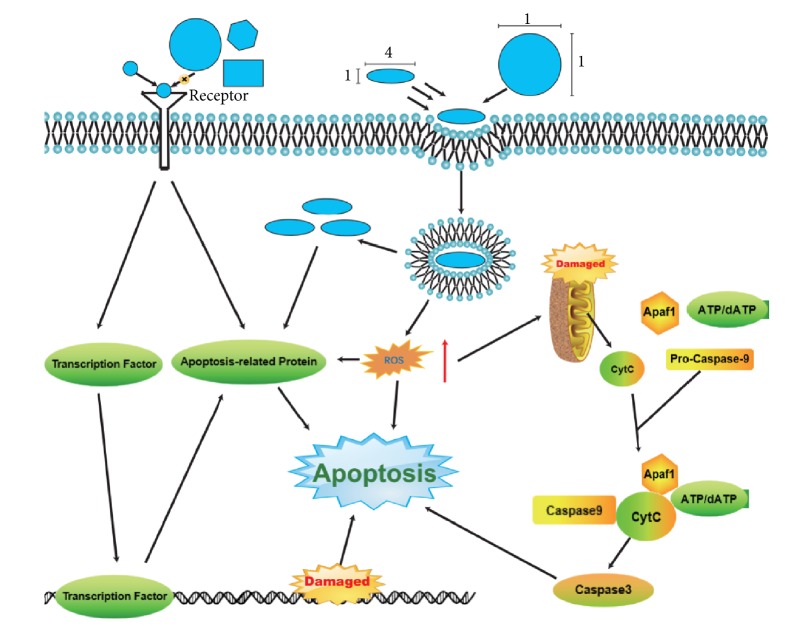
The effect of the shape of the nanoparticles on their action.

The shape of nanometer molecule is not a parameter that can be measured, but its influence on nanometer material molecule is self-evident. The European Union's new guidelines on nanomaterials state that shape is one of the key parameters in the definition of “nanoform” [22]. The US EPA is also considering shape as a condition for the registration and identification of nanoforms of special chemical substances [23]. All of the above illustrate the importance of shape as a parameter for nanomolecules, and graphene nanocomposite materials are no exception. The molecular structure of graphene nanocomposites is lamellar [24], which is considered by the authors as an advantage in terms of morphological parameters. It is not difficult to conclude from the basic physics that, compared with the spherical structure of fullerenes and the tubular structure of carbon nanotube materials, the “flake structure” of graphene can provide a larger specific surface area, which can make interactions between the surface of the molecule and its surroundings stronger [25]. Studies have shown that the interaction between NPs and biological units is strongly dependent on the shape of NPs [26, 27]. For example, the binding of NPs to the receptor as a ligand molecule is highly dependent on the shape of NPs [28]. Nanoscale shapes have also been shown to affect cellular absorption. Currently, there is a lack of concrete researches on the relationship between the specific morphology of graphene oxide nanocomposite molecules and their phagocytosis, which may be a complex problem because the relationship between the shape and cellular absorption of nanoparticles is “complex and diverse”. For example, experimental results have shown that the endocytosis of Au nanomaterials coated with transferrin is worse than that of spherical nanomaterials (two sizes:74 nm and 14 nm) [27, 29]. On the contrary, it has also been reported that in human melanoma cells (A375), compared with shorter silica nanorods (aspect ratio 2) and spherical silica nanoparticles (aspect ratio 1), elongated silica nanorods with aspect ratio 4 have higher internalization rate [30]. The effect of the shape of the nanoparticles on their action is illustrated in Figure 2. In the existing researches on GO nanocomposites, researchers pay more attention to the thickness and size of nanomaterial molecules and seldom discuss shape parameters such as aspect ratio. However, the above examples have demonstrated the importance of the specific shape of material molecules for their biological effects, so it is very necessary to obtain appropriate shape parameters for the use of nanomaterial molecules. In the future, it may be an important research direction to explore appropriate shape parameters through specific experiments.

#### 2.1.2. The Size of a Material Molecule

The molecular size of GO nanocomposites is one of the most important physical properties. Many unique properties of nanomaterials are determined by their molecular size. Because the size or diameter of nanomolecule is in the nanometer, the nanomolecule has a high surface area ratio and quantum size effect, so that the properties of nanomolecule have a significant difference compared with other common materials [31]. Many intrinsic physical properties of nanomaterials are affected by molecular size, such as superparamagnetism, plasmonic properties, and fluorescence properties [32–34]. By definition, GO with a lateral size range of 20 to 100 nanometers is known as nano-GO [4]. In some of the current experiments, the diameter of nano-GO used by the researchers is 50 nm-100nm [35]. However, we also found that in some experiments the researchers used materials with a molecular diameter of more than 100 nm [36, 37]. The photothermal effect of nanomolecule is related to the size of molecule. From the analysis of our existing experimental data, we can roughly infer that the photothermal effect of the material becomes worse with the increase of molecular diameter. The reason may be that with the increase of the molecular diameter of the material, the specific surface area decreases, and the actual light-receiving area of the material decreases, thus leading to the deterioration of the photothermal effect of the material. According to Roper's report, the calculation formula of photothermal conversion efficiency can support the conclusion above [38, 39].

(1) $$\begin{array}{c} \eta =\frac{hS\left(T_{max}-T_{surr}\right)-Q_{dis}}{I\left(1-10^{-A_{\lambda }}\right)} \end{array}$$

However, the specific relationship still needs further experimental exploration. In one study, Medintz and his colleagues confirmed that smaller gold nanoparticles (about 10 nm) were more likely to enter cells [40]. It has also been pointed out that uniform and appropriate nanomaterials less than 100 nm (especially 50-80 nm) are more suitable for drug delivery [41]. However, nanomolecules should not be too small in diameter since the amount of drugs they can carry is much smaller, which is not positive for treatments. Therefore, the molecular size of GO nanocomposites plays an important role in their functional realization. Adjusting the diameter of nanomolecules can give full play to the antitumor effect. The point here is that repeated oxidation can reduce the size of nanomolecules [42], which may have certain guiding significance to our future experiments.

The diameter of nanometer molecule can also affect its photodynamic effect. The application of photodynamic effect in antitumor therapy is a new way of thinking which is different from photothermal therapy [43]. Photodynamic therapy is a photochemical reaction produced by photosensitizers under specific light excitation and then could produce highly active singlet oxygen, which stimulates downstream signaling pathways and changes the biological behavior of cells. So far, traditional PSs such as methyl blue and rose bengal have been widely applied for PDT [44, 45]. However, the photodynamic efficiency of current photosensitizers is limited for the oxygen reliance, the light penetration depth in tissues, and so on [46, 47], which forces us to develop new photodynamic materials. In this context, GO nanomaterials are increasingly used in photodynamic therapy [48]. Photodynamic therapy can be better achieved through nanomolecules, mainly because of their controllable shape, size, and many unique functions [49–53]. As mentioned above, nanomaterials have large specific surface areas due to their proper shape and size. Studies have shown that nanomolecules with large specific surface areas can carry more photosensitive molecules into tumor tissues [54].

**Figure 3:**
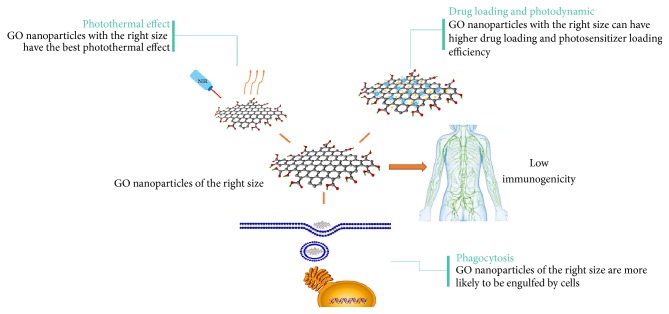
The importance of appropriate nanoparticle size to the application of GO.

Besides, the molecular size of nanomaterials not only plays an important role in the functional realization of nanomaterials, but also influences the toxicity of nanomaterials and the body's immune response to nanoparticles. Studies have shown that the toxicity of GO nanomolecules is related to their molecular size [55]. Schinwald et al. reported that large diameter GO molecules can cause inflammation in humans [56]. A study by Kiew et al. has shown that controlling the molecular size of about 150 nm can prevent macrophage recognition [57]. Therefore, it is necessary to determine the size of molecules in the research, and the most common method is the electron microscope. The importance of appropriate nanoparticle size is shown in Figure 3.

It should be pointed out that there are still some problems in determining the shape and size of GO nanoparticles. For example, proteins immediately attach themselves to nanomaterials after they come into contact with them, creating the so-called protein corona around the nanomaterials [58, 59]. The interaction of the nanoprotein or the formation of the protein corona in the serum or in any other protein solution will have a significant effect on the shape, size, and many other properties of the nanomaterials [60–63]. It is not only nanomolecules that are easy to combine with proteins, but also other biological macromolecules such as DNA, which produces a series of changes in shape and size and gives many biological functions [64]. Nanomolecules can also absorb small molecules, such as glucose and amino acids, which may be the substrate for some chemical reactions or the “key” to some reactions. When combined with these small molecules, the nanomolecules can form a “bridge” shape that facilitates certain chemical reactions [65]. Nanomolecules themselves are also agglomerative; the agglomeration of nanomolecules depends not only on their own van der Waals force and other properties but also on the dispersion medium [66]. For example, many nanomaterials are more likely to aggregate in electrolyte solutions, and we found this with GO in our experiments. After the phenomenon of protein adsorption or agglomeration, the actual particle size of the nanomolecule has changed, and its physical and chemical properties will be greatly affected. At present, some researches are devoted to solving the agglomeration problem of nanomolecules [67], but only in the aspect of engineering application. The problems mentioned above can occur with GO nanoparticles, so further exploration is needed to solve these problems in the aspect of medicine.

#### 2.1.3. Surface Potential of a Material Molecule

Surface potential is an important physical quantity of nanomolecules. We can detect the surface potential of nanomolecules through the measurement of surface zeta potential. Zeta potential of GO nanomolecules is one of the physical quantities frequently measured in experiments. One reason that zeta potential is particularly significant is that it could affect the dispersion of graphene oxide nanomolecules. Some studies indicate that particles with zeta potential between -30 and + 30mv tend to condense, which is most obvious when the solution is at an isoelectric point, when zeta potential of nanomolecule is 0mv [68]. Currently, in the preparation of GO, the stability of nanomolecular solution of GO is often enhanced by adsorption of polymer molecules on the surface. The surface properties of modified polymer molecules changed, and the surface potential changed accordingly, so that the modified polymer molecules could not be easily polymerized into macromolecules. Polymer adsorption is an effective surface modification method to improve the stability of drug suspensions [69]. The surface of GO nanomolecules is often modified with PEG or other macromolecules, and zeta potential is also mostly located outside the above condensation range. Measuring the zeta potential of the material molecule also helps to determine the isoelectric point of the material molecule, which is also a very important physical quantity of the material molecule. Therefore, it is necessary to measure the zeta potential of the material molecule [70, 71].

There are also many other physical properties of nanomolecules, such as superparamagnetism, porosity, and dust content. These physical quantities also have a great influence on the properties of nanomolecules, but these physical properties do not play a critical role in drug loading, photothermal effect, and photodynamic effect. Therefore, the authors believe that it is not necessary to measure the characterization of nanomolecules in detail in experiments. At present, most researches on nanomaterials have not reported many other properties of nanomaterials. With the in-depth studies of nanomolecules, more physical properties may be paid attention to in the future.

### 2.2. Chemical Properties of a Material Molecule

**Figure 4:**
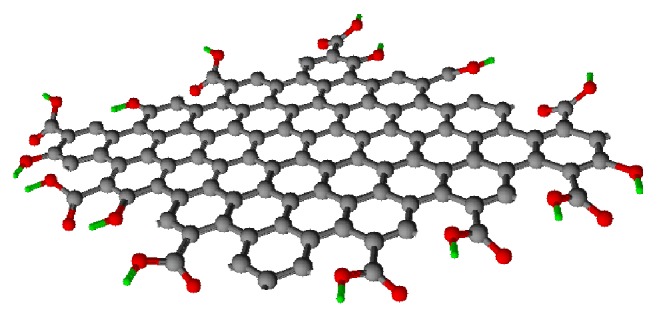
The structure of GO. In the picture, the black spheres represent carbon atoms, the red spheres represent oxygen atoms, and the green spheres represent hydrogen atoms.

The chemical properties of GO nanomolecules that we focus on are the surface chemical properties. The rich chemical properties of GO provide us with more opportunities for modification and drug delivery. As we all know, functionalized nanographene can be used as a drug carrier to deliver anticancer chemotherapy drugs and gene therapy for tumors in vitro [17, 72–74]. Meanwhile, graphene phototherapy for tumors has shown promise in many in vivo and in vitro studies, such as photothermal therapy [9] and photodynamic therapy [75]. However, before the graphene nanoparticles are used in the process of tumor treatment, we must modify functional groups on the surface of the material molecular. The first is the oxidation of graphene nanomolecules using oxidants such as potassium permanganate. Currently, the synthesized GO contains a large number of oxygen-containing functional groups, such as hydroxyl groups, aldehyde groups, and carboxyl groups, in its base plane and edges [76, 77]. The specific molecular structure of GO is shown in Figure 4.

The presence of these functional groups can transfer negative surface charges and inhibit irreversible lamination in solution [78], which enables the GO nanomolecules to have good hydrophilicity and water solubility [79], so that the GO nanomolecules are more compatible with the biological system, are absorbed and transported in the body efficiently, and achieve the purpose of treating diseases. In addition, the presence of these oxygen-containing functional groups facilitates further surface modification, providing possible sites for connecting other molecules [80], such as amidation [81]. However, with the number and types of surface groups increased, the electron cloud distribution of original graphene nanomolecules will change and the atoms of molecule will no longer be in a plane; as a result, many original functions related to large *π*-*π* bonds, such as drug carrying capacity, are probably affected. Therefore, it can be seen that the biological functions of GO nanoparticles are inseparable from their inherent surface chemical properties. These surface modifiers also play an important role in the photodynamics of GO nanoparticles [82].

However, graphene oxide nanomolecules are not suitable for biomedical applications if only due to their own chemical properties. Therefore, we often modify graphene nanomolecules with some molecules, such as chitosan, PEG, and albumin. The addition of these molecules will have a certain impact on the chemical properties of GO nanomolecules. Some representative modifying molecules are discussed in detail in the following subsections.

#### 2.2.1. Chemical Properties of GO Nanoparticles Grafted with PEG

**Figure 5:**
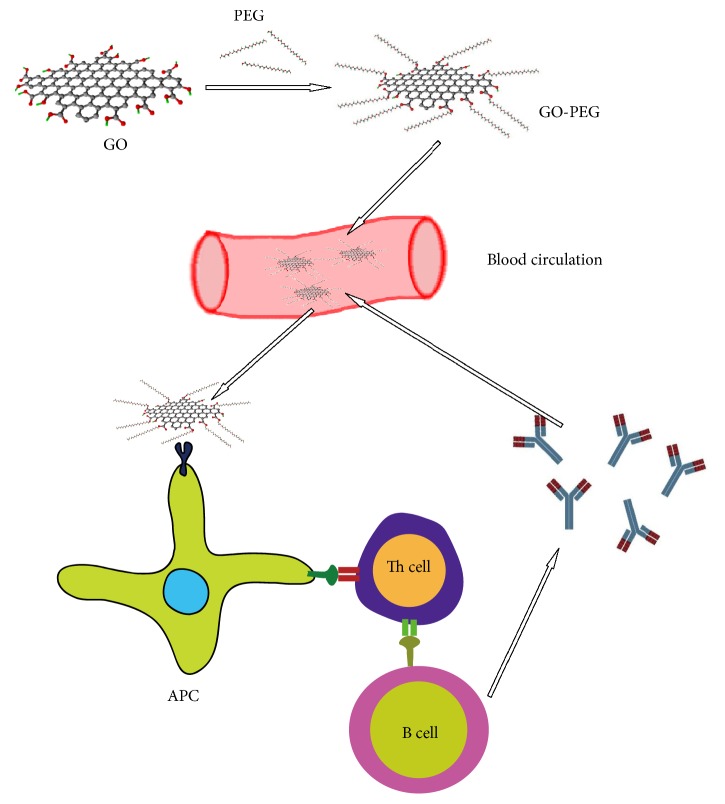
GO-PEG causes unnecessary immune responses in the body.

It is well known that the protein “corona” is easily attracted by nanomolecules when they enter the body due to hydrophobic, electrostatic, and van der Waals interactions, as well as the hydrogen bond between NPs and proteins [83–85]. This change may increase the clearance rate of nanoparticles in vivo [86, 87], may also affect the conformation of adsorbed proteins, thus inducing new epitope exposure, and may change the function of proteins, thus producing a series of contrary effects [59, 88]. Surface-functionalized polymers that make nanomolecules “stealth” are a reasonable solution [89]. So far, graphene oxide nanomolecules with polyethylene glycol have become the “golden standard” for surface functionalization in biomedical and engineering applications [90]. Polyethylene glycol on the surface of the material molecule can increase the hydrophilicity of polyethylene glycol, prevent the adsorption of excess proteins on the surface of the material molecule, and maintain good biocompatibility. However, PEG is not biodegradable, and the accumulation of pegylated drugs in the body can lead to the production of anti-PEG antibodies, which can accelerate the blood clearance rate and severe allergic reactions after administration [91–94].  Figure 5 illustrates this process. Therefore, it is necessary to explore new molecules that can make graphene oxide nanomolecules “stealth”.

#### 2.2.2. Chemical Properties of GO Nanomolecules Grafted with FA-BSA

**Figure 6:**
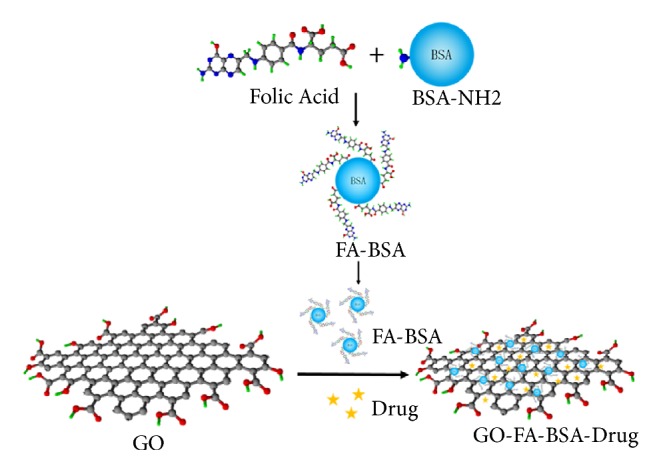
The synthesis and drug loading of GO-FA-BSA.

At present, antitumor chemotherapy drugs are generally cytotoxic. Taking adriamycin as an example, it has low tumor cell selectivity and strong cardiac toxicity [95–97]. Therefore, medical researchers hope to synthesize a cell-targeted drug-loaded nanoparticle by biologically modifying GO nanoparticles to be specifically identified, phagocytosed, or absorbed by tumor cells to kill tumor cells. The use of folic acid as a solution to this problem has become a common idea because the folic acid receptors on tumor cells are often overexpressed, which is much higher than in normal cells [98]. However, the direct linking of FA on GO often leads to physiological fluid aggregation, and the introduction of folic acid is often accompanied by the introduction of other stabilizers to stabilize the nanomolecules [17, 99, 100]. This functionalization is preferably noncovalent, as noncovalent functionalization has the advantages of reducing chemical reactions, reducing purification steps, and maintaining the original conjugated structure and physical properties of GO [101, 102]. Therefore, it is very important to design a noncovalent functional molecule of GO that can be used as both a stabilizer to prevent the aggregation of GO and a target to tumor cells. At present, a new active targeted drug carrier FA-BSA/GO system has been successfully designed.  Figure 6 briefly introduces the synthesis and drug loading of GO-FA-BSA. This system adopts folic acid grafted bovine serum albumin (FA-BSA) as stabilizer and target agent to improve the stability and dispersion of GO in physiological fluid, and good results have been achieved [103]. The authors believe that this idea may provide a better direction for the design and manufacture of antitumor graphene oxide nanomolecules in the future. When designing the molecular carrier, the stability of the drug and the carrier function in vivo should be taken into account, while the inherent properties of the drug molecule and the carrier molecule should be retained to maximize the antitumor effect and make it precise.

There are many other molecules that can be used to modify GO nanoparticles, such as chitosan, to perform different functions. Through chemical modification, we can subjectively modify graphene oxide nanomolecules to increase their functions, which is a highlight of graphene oxide applications.

## 3. Summary and Scope

With the development of medical technology, the cooperation between medicine, material science, and chemistry has become more and more, and the interdisciplinary has become a suitable choice to make a new breakthrough in the related fields. The application of graphene oxide nanomolecules in antitumor effects is a representative achievement of the intersection of medicine, materials science, and chemistry. GO nanoparticles are modifiable and have good photothermal and photodynamic effects, which provides a good idea for targeted killing of tumors. Nowadays, graphene oxide nanomolecules have been applied in many antitumor researches. Scientists have carried out various chemical modifications and physical properties control, loaded with different antitumor drugs to kill tumor cells, and achieved certain research results. In the process of application, we found that determining the chemical and physical properties of nanoparticles is a critical step. A lot of special properties of graphene oxide nanometer carrier, such as thermal effect and ability of carrying drugs, are all related to the physical and chemical properties of the nanoparticles, and a multitude of graphene oxide surface oxygen groups are left for us to modify, so as to enrich the nature of nanoparticles. It can be concluded that the development of graphene oxide nanomolecular applications will be based on the understanding, utilization, and expansion of its rich physical and chemical properties.
